# Supplementary material for: Asynchronous magnetic resonance elastography: Shear wave speed reconstruction using noise correlation of incoherent waves
Source: Magn Reson Med. 2022 Oct 27;89(3):990–1001. doi: 10.1002/mrm.29502 (PMC9792433; doi:10.1002/mrm.29502)
Supplement: Supplementary file 1 — DATA S1 MATLAB code used to generate the numerical simulations of Figure 1 [file MRM-89-990-s002.zip › k-Wave/helpfiles/getSpacedPoints.html]

getSpacedPoints :: Functions (k-Wave)


# getSpacedPoints

Create vector of log or linear spaced points.

## Syntax

```
points = getSpacedPoints(X1, X2)
points = getSpacedPoints(X1, X2, N)
points = getSpacedPoints(X1, X2, N, spacing)
```

## Description

`getSpacedPoints` generates a row vector of either logarithmically or linearly spaced points between `X1` and `X2`. When `spacing` is set to `'linear'`, the function is identical to the inbuilt `linspace` function. When `spacing` is set to `'log'`, the function is similar to the inbuilt `logspace` function, except that `X1` and `X2` define the start and end numbers, not decades. For logarithmically spaced points, `X1` must be > 0. If `N` < 2, `X2` is returned.

## Inputs

|  |  |
| --- | --- |
| `X1` | starting points value |
| `X2` | ending points value (where `X2` > `X1`) |

## Optional Inputs

|  |  |
| --- | --- |
| `N` | number of points in the vector (default = 100) |
| `spacing` | `'log'` or `'linear'` spaced values (default = `'linear'`) |

## Outputs

|  |  |
| --- | --- |
| `points` | row vector of equally spaced points |

## See Also

`linspace`, `logspace`
